# Food insecurity and disability among working-age and older adults

**DOI:** 10.1017/S1368980024000570

**Published:** 2024-02-26

**Authors:** Mia Hadfield-Spoor, Mauricio Avendano, Rachel Loopstra

**Affiliations:** 1 Department of Nutritional Sciences, Franklin Wilkins Building, King’s College London, 150 Stamford Street, London, UK; 2 Department of Epidemiology and Health Systems, Center for Primary Care and Public Health (Unisante), University of Lausanne, Lausanne, Switzerland; 3 Department of Public Health, Policy and Systems, University of Liverpool, Liverpool, UK

**Keywords:** Food insecurity, Disability, Age, Socio-economic factors

## Abstract

**Objective:**

To explore relationships between disability, food insecurity (FI) and age and examine how socio-economic factors impact risk of FI among disabled people in working and older age.

**Design:**

Logistic regression models used to analyse the contribution of socio-economic factors to gaps in risk of FI for disabled people. In models stratified into working and older age groups, differences in risk of FI for disabled and non-disabled people were examined by employment, education and assets.

**Setting:**

England, Wales and Northern Ireland, 2016 and 2018

**Participants:**

A representative sample of 6187 adults aged 16+, of whom 28 % were disabled, from the Food & You survey.

**Results:**

The gap in FI risk by disability status decreased as age increased. For ages 25–34 for disabled *v*. non-disabled people, risk of FI was 31 % (95 % CI 21–41 %) *v*. 10 % (8–12 %); at ages 45 to 54, it was 18 % (11–23 %) *v*. 7 % (5–8 %), and at ages 75+, there was no gap in risk. Accounting for socio-economic variables halved the gap in risk among working ages. However, among working-age adults, FI among disabled people in full-time work was 15 % (11–20 %) compared with only 7 % (6–9 %) among non-disabled people in full-time work. Among older people, disabled people without savings were at higher risk of FI (5 % (3–7 %)) than non-disabled people without savings (2 % (1–3 %)) but having savings closed risk gap.

**Conclusions:**

Socio-economic resources partially explain disparities in FI risk when disabled. Disparities remained for people in full-time work and among people without savings in older age.

Food insecurity (FI) is common in low-income countries, but it is also a critical and increasing public health concern in high-income countries^(1,2)^. The United States Department of Agriculture defines household FI as the uncertainty of having, or inability to acquire, enough food to meet the needs of all household members at all times in socially acceptable ways because of insufficient money or other resources for food^(3)^. Low food security is characterised by reduced dietary quality or variety of diet with possible indication of reduced food intake; very low food security is when there are multiple indications of disrupted eating patterns and reduced food intake^(3)^. Research in the UK, USA and Canada suggests the risk of FI increased during the COVID-19 pandemic and with rising costs of living^(4,5)^. Food insecurity monitoring by The Food Foundation using YouGov’s UK panel recently showed that from a monthly prevalence of around 7 % in January 2021, moderate and severe experiences of FI were much higher, around 18 %, in January 2023^(6)^.

Some groups have been identified as having systematically higher risk of FI. These include people in receipt of income-replacement social security, people who are unemployed or underemployed, adults in younger age groups and people from disadvantaged groups^(1,7–9)^. Similarly, disabled people have also been found to be at higher risk compared with non-disabled people across several high-income countries^(1,10–14)^. A recent study found that having multiple disabilities, as well as a combination of both physical and mental/cognitive disabilities, was associated with increased risk of moderate-to-severe and chronic FI, particularly among working-age adults^(15)^. However, the explanation of these differences remains unclear – in particular, whether these differences affect only working-age adults or also older adults and whether they result primarily from differences in socio-economic resources. Data from the UK consistently show that risk of FI declines with age and is particularly low among over 65s^(1,2)^. On the other hand, Census data from the UK show that about 42 % of State Pension age adults were disabled in 2021^(16)^. It is yet unclear how the relationship between disability and FI varies between working ages and older ages.

There are multiple reasons why the risk of FI associated with disability may not be present at older age. According to the biopsychosocial model of disability, disability is the result of an interaction between a person and their environment and social context, including their socio-economic position^(17,18)^. Thus, the experience of disablement may differ with the changes in socio-economic circumstances that tend to occur with ageing^(19,20)^. For example, financial security generally increases over the life course as individuals accumulate savings and assets. Additionally, social security (i.e. in the form of state pensions) tends to be more secure, and more services are provided for people of older age^(21–23)^. At working age, there is often a large gap in income between disabled and non-disabled people; disabled people are more likely to be in deep poverty and less likely to be in full-time employment^(24)^; and disabled people are more likely to have lower educational attainment, earnings and likelihood of home ownership than non-disabled people^(25)^. A disability-income gap may not be evident at older age, as sources of income are more homogenous between disabled and non-disabled groups (i.e. pension income). Further, disability becomes more prevalent at older age, affecting people from both low and high socio-economic groups. Yet, older people who have been disabled for a long time may not have built up private pensions, savings or accumulated wealth through home ownership due to cumulative disadvantage^(19)^, thus risk of FI may still be higher for some disabled older adults. Importantly, even when disabled people have the same socio-economic resources as non-disabled people, other factors such as problems with transport, higher costs of living and difficulties with food preparation may increase their risk of FI. Identifying which factors close the gap in risk of FI for disabled people is important for understanding potential points of intervention and identifying where additional risk factors need to be explored.

In this paper, we first examine how the risk of FI associated with disability changes across age bands (roughly 10 years each from age 16 to 75+). We then explore the contribution of socio-economic status, particularly work status, qualifications and wealth to this relationship. We expect that the higher risk of FI among disabled people will be reduced once we account for the higher likelihood of disabled people being socio-economically disadvantaged, especially at working age. Lastly, we explore where gaps in risk of FI remain between disabled and non-disabled in the same socio-economic groups and where the gap in risk closes, focusing on employment status, home ownership, access to savings and educational attainment. We stratify this analysis into working-age (16–64) and older age adults (65+) because of differences in employment status (i.e. pension age was 65 for men and women in 2018) and because, as highlighted above, socio-economic resources are more evenly distributed between disabled and non-disabled people in older age.

## Methods

Throughout this paper, we use the identity-first terminology of ‘disabled people’^(26)^, preferred by Disability Rights UK, who advised on the project in which this study was included.

### Data source and sample

Data came from two waves of the Food Standards Agency’s Food & You survey (F&Y), a repeat cross-sectional, representative survey of adults aged 16 and over in England, Wales and Northern Ireland. The survey used random probability sampling and face-to-face computer-assisted personal interviewing. At the time that analysis began, it was the only nationally representative dataset in the UK containing an internationally agreed measure of household FI: the USDA’s Adult Food Security Survey Module^(27)^. Data from Wave 4 and 5 of F&Y, conducted in 2016 and 2018, respectively, were used. These independent samples were combined, resulting in a sample of 6187 adults^(28,29)^ of whom 28 % (*n* 1699) were disabled. Notably, these data were collected from a relatively stable period in the UK and prior to the pension age changing from 65 to 66 for both men and women. They were also collected prior to the COVID-19 pandemic subsequent period of rising inflation, when relationships between disability, age and FI may have been fluctuating^(6)^.

### Survey measures

The operationalisation of disability differed slightly between the two survey waves. Wave 4 asked respondents if they had any physical or mental health conditions or illnesses lasting or expected to last for 12 months or more. If respondents answered yes, this was followed by a question asking whether the condition or illness reduces respondents’ ability to carry-out day-to-day activities a lot, a little or not at all, in line with the Equality Act definition of disability and used in Office of National Statistics surveys. In wave 5, respondents were asked the same initial question but if respondents answered yes, the following question asked whether any of the conditions or illnesses affected respondents in specified domains. The domains listed were: vision, hearing, mobility, dexterity, learning/understanding/concentrating, memory, mental health, stamina/breathing/fatigue and socially/behaviourally. We merged this disability data by creating a new variable that combined people from Wave 4 who answered yes and who had a condition that reduced their ability to carry out day-to-day activities (a little or a lot) with people who in Wave 5 answered yes and reported at least one condition, illness or impairment. A sensitivity analysis was run to test whether use of one or the other measure changed the results.

FI was measured by the USDA’s 10-item Adult Food Security module, a validated scale that aims to capture prevalence of FI, at the household level, in the general population^(27)^. According to standard USDA practice, FI is identified by three or more affirmative responses to questions on the module. We use this binary measure of FI, capturing people with both low and very low food security.

### Covariates

The dataset provided age data in the following bands: 16–24, 25–34, 35–44, 45–54, 55–64, 65–74 and 75+. Gender was provided as a binary variable (male/female), as was presence of dependent child(ren) in the household (yes/no) and ethnicity (white ethnicity/other ethnicity). Marital status captured whether respondents were in marriage/civil partnership, single, separated, divorced or widowed. Data on education denoted whether a degree was the highest level of qualification a respondent achieved, another type of qualification or no qualification. Gross household annual income was only available in four income bands: <£10 399, £10 400–£25 999, £26 000–51 999 and >£52 000, as well as missing. Main employment status for the household was captured as a 9-level variable denoting: full-time education, paid employment, self-employed, unemployed, temporarily unable to work, permanently unable to work, retired, looking after the home, or other. Home ownership recorded the tenure of respondents’ living accommodation: own home outright, buying with a mortgage, renting or living rent free. Sixteen different sources of income data were captured including state and private sources. These were not mutually exclusive categories. The source of interest for our analysis was whether they collected interest from savings and investments because this income source represents a marker of wealth and access to assets, which could act as a financial security buffer^(30)^.

Low cell counts for some subcategories meant we had to reclassify some variables for descriptive and regression analyses. A binary housing tenure variable was made to capture households who had investment in their own homes (owned outright or buying on a mortgage) compared with people who were renting. Marital status was recoded into living with a partner or not living with a partner. For our stratified analysis of working-age adults (see below), we wanted to explore if people who were in the same work status group (e.g. unemployed) had similar risk of FI, whether disabled or not. To do this, we combined information about the nature of employment in the household (full-time or part-time) with employment status to denote household work status as (1) full-time work; (2) part-time work; (3) unemployed, temporarily inability to work, or waiting to take up work; (4) permanent inability to work and (5) retirement, in education, caring for the home/family or not working for other reasons. We had to combine reasons for being out of work for the latter group due to small numbers for these subgroups across disabled and non-disabled working-age adults.

With the exception of the income variable, data were missing for only 48 respondents; these individuals were excluded from the analysis. As 23 % of respondents had missing values for income, we included these individuals into the analysis, including an indicator variable for missing income in the analysis.

### Statistical analysis

First, to visualise the relationship between disability and FI across age bands, we used logistic regression including an interaction term for age and disability and corresponding predicted probabilities to examine risk of FI for disabled and non-disabled people by age bands (16–24; 25–34; 35–44; 45–54; 55–64; 65–74 and 75+).

Adding to this logistic regression model and including all survey respondents, we then added gender and ethnicity terms, followed by a model that added socio-economic characteristics, namely, qualification level, household income, main household employment status, housing tenure, presence of child(ren) in the household and partnership status. In Fig. 2, we plot the marginal difference in predicted risk of FI between disabled and non-disabled adults over age bands before and after adjustment for socio-economic characteristics to show how the risk gap for FI for disabled people changes. The results for the logistic regression models underlying this figure can be seen in Web Appendix Table A1.

Next, in models stratified into working-age and older age groups, we examined if differences in risk of FI were observed for adults in the same socio-economic subgroups or if there was evidence of gaps in risk of FI remaining. Among working-age adults, we examined differences in FI for disabled and non-disabled people by three markers of socio-economic status: main household employment status, highest qualification and housing tenure. Too few disabled people had savings to enable us to examine the impact of this asset on this relationship for working-age people. Then among older age adults, having already observed no difference in risk of FI between disabled and non-disabled adults in older age, we examine if any disparity in risk of FI is apparent for disabled older adults who were socio-economically disadvantaged compared with people who were not. We used information on savings and investments, highest qualification and housing tenure as markers for socio-economic advantage in older age.

## Results

### Descriptive statistics

In the combined F&Y Wave 4 and 5 sample, over one-fifth of respondents (21 %) were identified as disabled. In Table 1, we show characteristics of disabled and non-disabled people stratified into working-age and older age groups. In both groups, there were significant differences across socio-economic characteristics, with disabled people more likely to be in socio-economically disadvantaged groups. For example, among both working-age and older adults, disabled people were more likely to have no degree qualification than non-disabled adults (17 % *v*. 10 % for working-age; 42 % *v*. 28 % for older ages; for both, *P* < 0·0000). Among older adults, 75 % of disabled people owned their own home outright or were buying it compared with 86 % among non-disabled people and 24 % of disabled people were renting compared with 13 % of non-disabled people (*P* < 0·0003). Among working-age adults, 47 % of disabled people owned or were buying a home compared with 63 % of non-disabled people, and 51 % of disabled people were renting compared with only 34 % of non-disabled people (*P* < 0·0000). Among working-age adults, only 52 % of disabled people were in households with paid employment compared with 73 % of non-disabled people (*P* < 0·0000). However, there was no difference in whether households had earnings from savings between disabled and non-disabled among both working-age and older age adults (*P* > 0·05 for both age groups).

**Table 1:** Socio-economic characteristics of disabled and non-disabled people stratified by working and older age

	Under 65			Over 65		
	Disabled (*n* 911)	Non-disabled (*n* 3363)	*P* value for *X* ^2^	Disabled (*n* 786)	Non-disabled (*n* 1101)	*P* value for *X* ^2^
**Sex**			0·0174			0·0296
Female	55 %	49 %		58 %	52 %	
Male	45 %	51 %		42 %	48 %	
**Highest qualification**			*P* < 0·0000			*P* < 0·0000
Degree	25 %	37 %		17 %	24 %	
Other	58 %	53 %		41 %	48 %	
None	17 %	10 %		42 %	28 %	
**Household main employment status**			*P* < 0·0000			0·0061
Full-time education/training	1 %	2 %		0 %	8·0e-04 %	
In paid employment	52 %	73 %		6 %	12 %	
Self-employed	12 %	12 %		5 %	6 %	
Unemployed or waiting to take up work	2 %	1 %		0 %	3·1e-04 %	
Temporarily unable to work	4 %	0 %		0 %	7·4e-04 %	
Permanently unable to work	13 %	1 %		2 %	0 %	
Retired	7 %	5 %		85 %	80 %	
Looking after the home	8 %	4 %		1 %	1 %	
Doing something else	1 %	1 %		1 %	1 %	
**Work status**			*P* < 0·0000			0·0004
Full-time work	50 %	75 %		4 %	10 %	
Part-time work	14 %	10 %		8 %	7 %	
Waiting to take up work, unemployed and temporarily unable to work	6 %	1 %		0 %	0 %	
Permanently unable to work	13 %	1 %		2 %	0 %	
Retired and not working for other reasons	17 %	12 %		86 %	83 %	
**Household income band**			*P* < 0·0000			0·0011
<£10 399	11 %	4 %		10 %	7 %	
£10 400-£25 999	26 %	15 %		33 %	30 %	
£26 000-£51 999	19 %	25 %		19 %	24 %	
>£52 000	18 %	29 %		8 %	14 %	
Missing	25 %	27 %		30 %	26 %	
**Marital status**			*P* < 0·0000			*P* < 0·0001
Single	42 %	44 %		7 %	7 %	
Married/civil partnership	41 %	47 %		53 %	63 %	
Separated	3 %	2 %		1 %	2 %	
Divorced	11 %	5 %		10 %	9 %	
Widowed	3 %	1 %		29 %	18 %	
**Dependent children in household**			0·5928			0·9104
Yes	44 %	46 %		9 %	9 %	
No	56 %	54 %		91 %	91 %	
**Home ownership**			*P* < 0·0000			*P* < 0·0003
Own it outright	21 %	22 %		72 %	82 %	
Buying with help of mortgage/loan	26 %	41 %		3 %	4 %	
Part own and part rent	0 %	1 %		0 %	0 %	
Rent	51 %	34 %		24 %	13 %	
Live here rent free	2 %	2 %		1 %	1 %	
**Household earnings from savings and investment**			0·7807			0·2358
Yes	7 %	7 %		16 %	18 %	
No	93 %	93 %		84 %	82 %	

### Food insecurity risk by disability status and age band

**Fig. 1:**
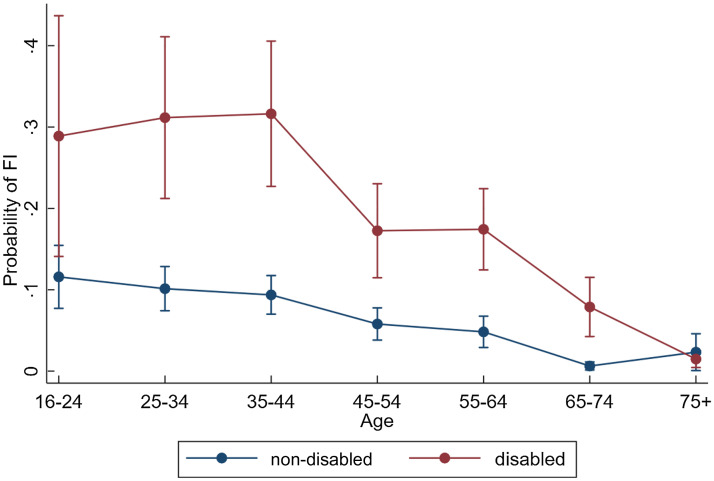
Probability of food insecurity for disabled and non-disabled people at different ages (unadjusted)

In Fig. 1, we show the risk of FI by age band for disabled and non-disabled adults. The gap in FI risk by disability status decreased as age increased. There was a wide gap in risk until about age 45 (though confidence intervals were wide for the 16–24 age group). For ages 25–34 for disabled *v*. non-disabled people, predicted risk of FI was 31 % (95 % CI 21–41 %) *v*. 10 % (95 % CI 8–12 %), a risk gap of 21 percentage points. From age 45, the gap in risk of FI appeared to reduce between disabled and non-disabled people. For ages 45 to 54, the predicted probability was 18 % (95 % CI 11–23 %) *v*. 7 % (95 % CI 5–8 %) for disabled *v*. non-disabled adults, a risk gap of only 11 percentage points. The gap between disabled and non-disabled people then closed further at age 65–74, and by age 75+, there was no visible difference in risk of FI between disabled and non-disabled adults.

### Contribution of socioeconomic factors to food insecurity disparities

**Fig. 2:**
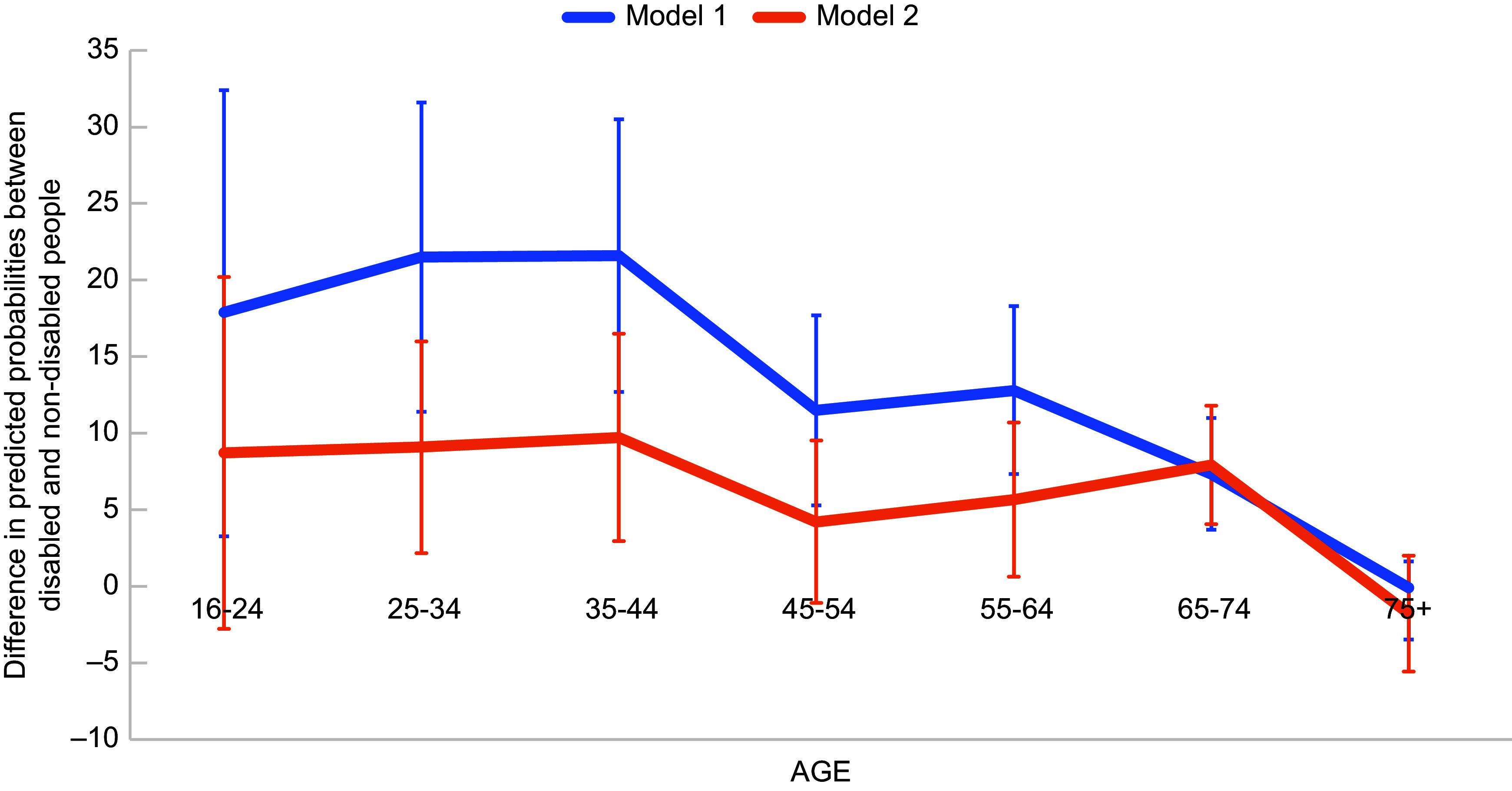
Impact of adjusting for demographic and socio-economic factors on differences in predicted probability of food insecurity between disabled and non-disabled people. Notes: Model 1 is adjusted for gender and ethnicity. Model 2 is additionally adjusted for highest level of qualification, employment status, household income, presence of children in the household, home ownership and partnership status

In Fig. 2, we show the plotted risk gaps (i.e. differences in predicted probabilities) between disabled and non-disabled adults before and after adding socio-economic characteristics to a model adjusted for gender and ethnicity. In model 1, we see that the gap in risk of FI by disability status is 21 percentage points (95 % CI 12–31 %) for the 25–34 age bands, 12 percentage points (95 % CI 5–18 %) for ages 45–54 and 13 percentage points (95 % CI 7–18 %) for 55–64, compared with 7 percentage points (95 % CI 4–11 %) for age band 65–74 and close to zero for adults aged 75+. For all working-age bands, the addition of socio-economic variables to the model reduced the difference in risk of FI between disabled and non-disabled people by about half. For example, the 21 percentage point difference in FI at ages 25–34 between disabled and non-disabled people declined to a 9 percentage point difference (95 % CI 3–16 %).

### Do employment status, housing tenure and/or education close gaps in risk of food insecurity for disabled people of working age?

**Fig. 3:**
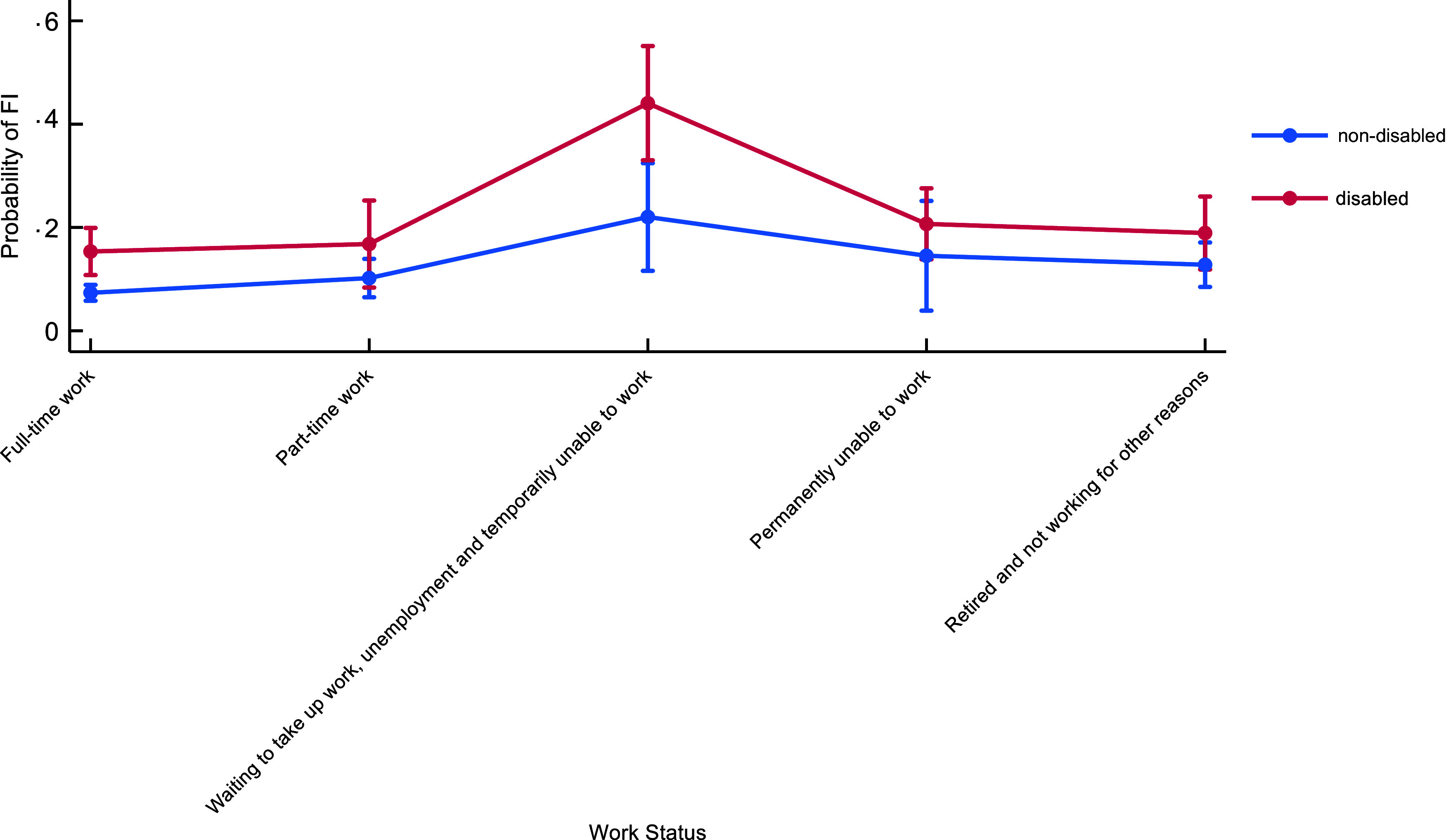
Adjusted predicted probability of food insecurity among working age disabled and non-disabled adults by household work status. Note: Predicted probabilities from a logistic regression model adjusted for sex, ethnicity, highest qualification, household savings, presence of child(ren) in household, household income, homeownership and presence of partner

Figure 3 shows predicted probabilities of FI by disability status and household work status among working-age adults. Though full-time work reduced the risk of FI for both disabled and non-disabled people, the risk of FI among disabled people in households with full-time work remained significantly higher than non-disabled people: 15 % (95 % CI 11–20 %) compared with the 7 % (95 % CI 6–9 %) for non-disabled people in households with full-time work. There was also a significantly higher risk of FI among disabled people who were ‘unemployed, waiting to take up work or temporarily unable to work’ compared with non-disabled people with this status. However, there was no significant difference in risk of FI for people who were in part-time work, permanently unable to work or not working for other reasons.

Having no degree level qualification equalised risk of FI between disabled and non-disabled working-age adults (web appendix Figure A1). Among people with degree-level qualification, the predicted risk of FI among disabled people was higher at 14 % (95 % CI 8–19 %) compared with 7 % (95 % CI 4–9 %) for non-disabled people, though confidence intervals overlapped. There was also a significant difference in risk of FI between disabled and non-disabled working adults with some qualification but not a degree.

Home ownership also may not equalise the risk of FI between disabled and non-disabled working-age adults, with predicted probability of FI for disabled adults at 9 % (95 % CI 6–13 %) compared with 4 % (95 % CI 3–6 %) for non-disabled adults (web appendix Figure A2), but did appear to reduce the gap compared with people living in rental accommodation. Here, the predicted probability of FI was 24 % (95 % CI 19–30 %) among disabled adults *v*. 15 % (95 % CI 12–17 %) among non-disabled adults.

### Is economic vulnerability in older age associated with higher risk of food insecurity for disabled older age adults compared with non-disabled older age adults?

**Fig. 4:**
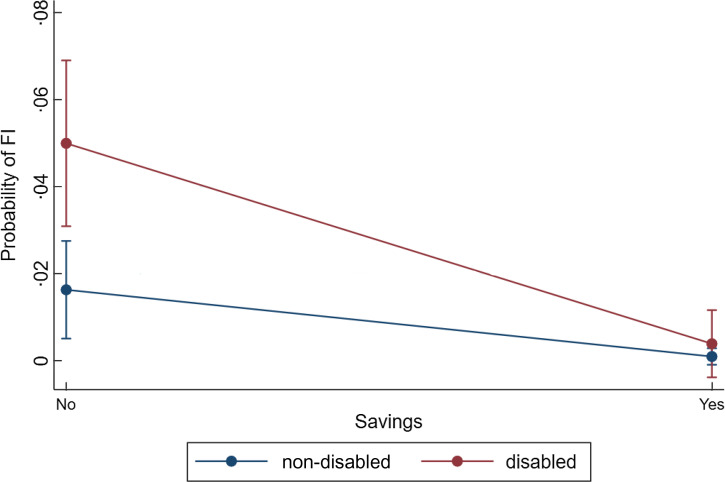
Adjusted predicted probability of food insecurity among older disabled and non-disbaled adults by access to savings. Note: Predicted probabilities from a logistic regression model adjusted for sex, ethnicity, highest qualification, presence of child(ren) in household, household income, homeownership and presence of partner (work status not included for 65+)

Figure 4 shows the predicted probabilities of FI by disability status and savings for older adults. Whilst the overall probability of FI was low for all older age adults, among disabled people who had no savings, the predicted level of FI was close to 5 % (95 % CI 3–7 %), significantly higher than non-disabled older adults without savings (2 % (95 % CI 0·5–3 %). In contrast, savings appeared to close the gap in risk of FI for older age adults, with no difference in risk of FI between disabled and non-disabled people.

Home ownership also appeared to narrow the gap in risk of FI for older disabled compared with non-disabled adults (web appendix figure A4). Disabled people who were renting had a predicted risk of FI of about 7 % (95 % CI 2–11 %) compared with only about 1 % (95 % CI 0–2 %) for non-disabled people. Among homeowners, the gap was only about 2 percentage points and differences in risk were not statistically significant.

There were NS differences in risk of FI between disabled and non-disabled people at older age among people with a degree qualification or other qualification; indeed, the risk of FI for both disabled and non-disabled adults with degree qualifications was not significantly different than zero (web appendix figure A3). However, among people with no qualification, the risk of FI was significantly higher (7 % (95 % CI 3–12 %) *v*. 2 % (95 % CI 0–4 %)).

### Sensitivity analyses

In sensitivity analyses, we implemented models examining the interaction between FI and age band using data for the 2016 and 2018 surveys separately, given that disability was measured differently across these two years. Our results were consistent across survey waves, albeit with wider confidence intervals, suggesting that the difference in classification of disability between survey waves did not change relationships between disability and age in relation to FI (Web Appendix Figure A4).

## Discussion

In this paper, we explored differences in the probability of FI between disabled and non-disabled people across different age bands. We found that gap in FI risk was largest between disabled and non-disabled people among people under 45, and that it closed for adults aged 75 and older. At working ages, socio-economic factors explained about half of the difference in risk and appeared to eliminate the observable gaps in risk for adults 65+. In stratified models for working-age and older age adults, we observed where there were gaps in risk of FI between disabled and non-disabled people in the same socio-economic group and where these gaps were closed. We observed that significant gaps in risk remained between disabled and non-disabled working-age adults among people where the main earner had full-time work and where the main earner was unemployed or temporarily unable to work. Gaps in risk were NS where main earners were permanently unable to work or not working for other reasons. Having a degree qualification or other qualification also did not close the gap in risk of FI between disabled and non-disabled working-age adults, though among people with no education, risks were the same among disabled and non-disabled adults. Lastly, there were significant differences in risk between disabled and non-disabled among renters and among homeowners, though gaps were narrower for the latter group. Among older age adults, it was disabled people who were in more disadvantaged groups that had significantly higher risk of FI, namely, people who were without savings, without a qualification and were renting their home. Having savings in older age closed the gap in risk of FI between disabled and non-disabled adults.

Our results are consistent with research from other high-income countries, which have found that disabled people generally have higher risk of FI than non-disabled people as a result of disadvantage^(13,31)^. Previous research has also suggested that FI decreases with age^(31,32)^, but that some groups of older people such disabled people and who are disadvantaged may still be at higher risk of FI at older ages^(7,21)^. Our findings also support research that indicates that wealth and assets such as savings are particularly important for disabled peoples’ food security^(33)^; indeed, savings eliminated the difference in FI risk between disabled and non-disabled people in older age. Savings may be particularly beneficial for disabled people who can experience higher day-to-day living costs, such as energy costs, travel costs and care costs^(34)^. Unfortunately, due to low numbers of working age people with savings, we could not confirm if the same was true for disabled people of working age.

The high-level finding that the gap in risk of FI between disabled and non-disabled adults declines with age suggests age may be protective against disparities in FI risk associated with disability. This may reflect the relatively higher level of protection against economic disparities for older people in the UK as a result of pensions and other financial supports. For example, the ability to access state pension, which is more generous than social security for people unable to work, may lead to greater economic security among both disabled and non-disabled people of pensionable age^(35)^. It may also reflect other forms of social support and services that may impact on food security beyond socio-economic factors including free public transport, access to social services and activities providing free or low-cost meals for older people^(23)^. Targeted financial support for older people that we were unable to capture in our analysis may also contribute to greater food security in older age for disabled people, for example, free prescriptions and winter fuel allowance. Another explanation for the high-level finding is that many people become disabled in older age, and therefore may be socio-economically better off compared with younger disabled people^(35)^. Whilst we could not examine this hypothesis directly due to the cross-sectional nature of our data and lack of information on duration of disability, our analysis of disability and FI in older age suggested that disabled people who were better off socio-economically had no difference in risk of FI from non-disabled people, but that gaps in risk were apparent for disabled people from lower socio-economic backgrounds (i.e. no qualification; renting their home; lacking savings). These findings suggest the benefits of older age may not equally reach people who are disabled or that further support is needed to meet their food needs. For example, physically accessing food and preparing it may be more difficult for more severely disabled older adults compared with non-disabled adults^(36)^, particularly where both lack financial assets. A final explanation for the reduction in risk gap between disabled and non-disabled adults among people aged 75+ that cannot be ruled out is selective survival, as research has found that disability is associated with increased mortality^(37–39)^ a different demographic composition of disabled people at older ages, however, this needs examining in longitudinal data.

Among working-age adults, we observed that socio-economic factors explained some difference in risk between disabled and non-disabled people, however, about 50 % of the risk gap remained. In our working-age models, we observed persistent gaps in risk of FI between disabled and non-disabled people remained among people with full-time work and people who were unemployed or temporarily unable to work. Similarly, having a degree qualification or other qualification and home ownership did not close the gap in risk between disabled and non-disabled people, and disabled people who were renting had a much higher risk of FI compared with non-disabled people who were renting.

These findings suggest unobserved factors may play a role. Among disabled renters, inappropriate accommodation for disabled people may impact on health and make it particularly difficult for people to access, store and prepare food, compared with non-disabled people. There are also higher costs of living associated with being disabled and with accessing food^(40)^. Experiences of discrimination may also make it harder for disabled people to go out to access food. Among disabled people in full-time work, work may be of poorer quality and pay may be lower for disabled people; disabled people are also more likely to experience job insecurity^(19,34,41,42)^. Our findings may also reflect that higher education may not translate into higher incomes for disabled people in the same way that it does for non-disabled people, similar to other stigmatised and marginalised groups^(7,8,43)^. These findings raise concerns about efficacy of work alone as a solution to poverty and FI among disabled people.

### Strengths and limitations

A strength of this study is the use of standardised measures of FI and disability and use of from a representative sample of UK adults. These data were collected at a time of relative stability in levels of FI in the UK; relationships between disability, age and FI likely fluctuated over the COVID-19 pandemic and subsequent rises in costs of living. There is a need for further examination of these relationships using more recent data. A relatively small sample size also limited our ability to examine type and severity of disability may influence relationships with age and FI. We also lacked data on age of onset of disability which would have been helpful for understanding how economic disadvantages of disability may accrue over working age and into older age. Instead, we used markers of socio-economic status more relevant in older age, namely savings and home ownership, in order to identify economically disadvantaged older disabled people. We are unable to establish, however, whether these factors reflect economic disadvantage since early age.

Our measure of FI is focused on financial access to food and therefore may underestimate the level of FI among disabled people who face non-financial challenges to accessing food^(44,45)^. Factors like ability to go out to purchase, transport and prepare food were not available in the dataset, which may influence FI among disabled people. These findings clearly highlight the need for more in-depth research that explores the mechanisms contributing to insecure access to food among disabled people. Our measure of household income was crude, and therefore these findings do not rule out low levels of income as one explanation. We also had only a crude measure of saving and investment, a binary variable indicating whether the respondent’s household was receiving interest from either of these sources. More detailed data on the value and nature of savings and investment would aid understanding of how these variables may reduce risk of FI. Because of having limited measures of socio-economic factors, we are unable to tell if having savings and owning a home reduce the risk of FI themselves or whether they may reflect cumulative financial characteristics we were unable to assess. In addition, we had no data on living costs, including housing costs or costs associated with living with a disability; data on these types of factors would have contributed to a better understanding of socio-economic differences in risk of disability. Future analyses would benefit from larger datasets with more detailed information on disability and FI, including measures capturing insecure food access arising from inaccessibility. Longitudinal assessments of disability and FI over the life course would also help better understand these relationships.

### Conclusion

Our findings suggest that socio-economic resources play an important role in the relationship between FI and disability, both at working ages as well as at older ages. Socio-economic factors explained about half of the relationship at working-age and more fully the relationship among older people. However, full-time work and having a degree qualification did not close the gap in risk of FI between disabled and non-disabled people, suggesting these factors are not sufficient to reduce disparities in FI between disabled and non-disabled people. Unobserved factors that contribute to disabled people’s increased risk of FI require further research. Our results suggest that targeting interventions to specific groups of disabled people, such as people living in rental accommodations, people in full-time work and older people without access to savings, may be effective in addressing the increased risk of FI associated with disability.

### Key findings

- Disparities in risk of FI between by disability status decrease with age and are close to zero at ages 75+.
- Socio-economic factors explain about half of the gap in predicted FI risk among working-age adults (16–64).
- We find that disabled people have higher risk of FI even among people in full time work, suggesting work itself may not be sufficient to reduce the gap in FI risk between disabled and non-disabled people.
- Among people 65+, savings and home ownership closed the gap in risk FI between disabled and non-disabled people.

## Supporting information

Hadfield-Spoor et al. supplementary material Hadfield-Spoor et al. supplementary material

## Supplementary material

For supplementary material accompanying this paper visit https://doi.org/10.1017/S1368980024000570.
