# Supplementary material for: Food insecurity and disability among working-age and older adults
Source: Public Health Nutr. 2024 Feb 26;27(1):e84. doi: 10.1017/S1368980024000570 (PMC10966857; doi:10.1017/S1368980024000570)

**Web appendix - Table A1 -** Logistic regression models (unadjusted, model 1, model 2), containing age*disability interaction term.

|  |  | Unadjusted Model | | Model 1 | | Model 2 | |
| --- | --- | --- | --- | --- | --- | --- | --- |
|  |  | OR | 95% CI | OR | 95% CI | OR | 95% CI |
| Disability | |  |  |  |  |  |  |
|  | Yes | 3.10 | (1.41-6.84) | 3.31 | (1.51-7.27) | 2.31 | (0.40-6.01) |
| Age | |  |  |  |  |  |  |
|  | 25-34 | 0.86 | (0.53-1.39) | 0.89 | (0.55-1.44) | 0.71 | (0.40-1.27) |
|  | 35-44 | 0.79 | (0.51-1.22) | 0.81 | (0.52-1.25) | 0.84 | (0.49-1.44) |
|  | 45-54 | 0.47 | (0.29-0.77) | 0.49 | (0.30-0.81) | 0.62 | (0.34-1.15) |
|  | 55-64 | 0.39 | (0.22-0.67) | 0.43 | (0.24-0.75) | 0.66 | (0.36-1.18) |
|  | 65-74 | 0.05 | (0.02-0.12) | 0.05 | (0.02-0.13) | 0.09 | (0.03-0.23) |
|  | 75+ | 0.18 | (0.06-0.54) | 0.20 | (0.07-0.60) | 0.29 | (0.09-0.95) |
| Disability # Age | |  |  |  |  |  |  |
|  | Disabled # 25-34 | 1.29 | (0.51-3.28) | 1.27 | (0.50-3.22) | 1.18 | (0.40-3.51) |
|  | Disabled # 35-44 | 1.44 | (0.57-3.67) | 1.36 | (0.54-3.41) | 1.16 | (0.38-3.51) |
|  | Disabled # 45-54 | 1.09 | (0.42-2.87) | 1.03 | (0.39-2.74) | 0.77 | (0.24-2.47) |
|  | Disabled # 55-64 | 1.34 | (0.52-3.48) | 1.25 | (0.48-3.24) | 0.88 | (0.29-2.71) |
|  | Disabled # 65-74 | 4.37 | (1.38-13.8) | 3.93 | (1.24-12.4) | 3.96 | (1.12-14.0) |
|  | Disabled # 75+ | 0.20 | (0.47-0.88) | 0.19 | (0.43-0.81) | 0.22 | (0.45-1.08) |
| Sex | |  |  |  |  |  |  |
|  | Female |  |  | 1.45 | (1.09-1.92) | 1.24 | (0.91-1.69) |
| Ethnicity | |  |  |  |  |  |  |
|  | Other |  |  | 1.63 | (1.10-2.42) | 1.47 | (0.96-2.26) |
| Child in HH | |  |  |  |  |  |  |
|  | No |  |  |  |  | 0.61 | (0.43-0.86) |
| Highest Qualification | |  |  |  |  |  |  |
|  | Other |  |  |  |  | 1.71 | (1.16-2.51) |
|  | None |  |  |  |  | 2.33 | (1.47-3.68) |
| HH income | |  |  |  |  |  |  |
|  | <£10,399 |  |  |  |  | 1.58 | (0.95-2.63) |
|  | £10,400-£25,999 |  |  |  |  | 1.55 | (1.06-2.27) |
|  | >£52,000 |  |  |  |  | 0.49 | (0.29-0.81) |
|  | Missing |  |  |  |  | 0.95 | (0.63-1.43) |
| Employment Status | |  |  |  |  |  |  |
|  | In paid employment |  |  |  |  | 1.10 | (0.33-3.71) |
|  | Self-employed |  |  |  |  | 0.96 | (0.27-3.46) |
|  | Waiting to take up work |  |  |  |  | 4.78 | (1.17-19.5) |
|  | Temporarily unable to work |  |  |  |  | 4.84 | (1.19-19.6) |
|  | Permanently unable to work |  |  |  |  | 1.87 | (0.54-6.55) |
|  | Retired |  |  |  |  | 1.01 | (0.28-3.58) |
|  | Looking after the home or |  |  |  |  | 2.34 | (0.63-8.68) |
|  | Doing something else |  |  |  |  | 0.92 | (0.21-4.01) |
| Homeownership | |  |  |  |  |  |  |
|  | Renting |  |  |  |  | 3.55 | (2.51-5.02) |
| Living with partner | |  |  |  |  |  |  |
|  | Yes |  |  |  |  | 0.79 | (0.61-1.04) |

**Figure A1** Adjusted predicted probability of food insecurity among working-age disabled and non-disabled adults by highest qualification.

Note: Predicted probabilities from a logistic regression model adjusted for sex, ethnicity, presence of child(ren) in household, household income, work status, household savings, presence of partner and home ownership.

**Figure A2** Adjusted predicted probability of food insecurity among working-age disabled and non-disabled adults by home ownership.

Note: Predicted probabilities from a logistic regression model adjusted for sex, ethnicity, presence of child(ren) in household, household income, work status, household savings, and presence of partner.

**Figure A3** Adjusted predicted probability of food insecurity among older age disabled and non-disabled adults by highest qualification.

Note: Predicted probabilities from a logistic regression model adjusted for sex, ethnicity, presence of child(ren) in household, household income, household savings, presence of partner and home ownership.

**Figure A4** Adjusted predicted probability of food insecurity among older disabled and non-disabled adults by home ownership.


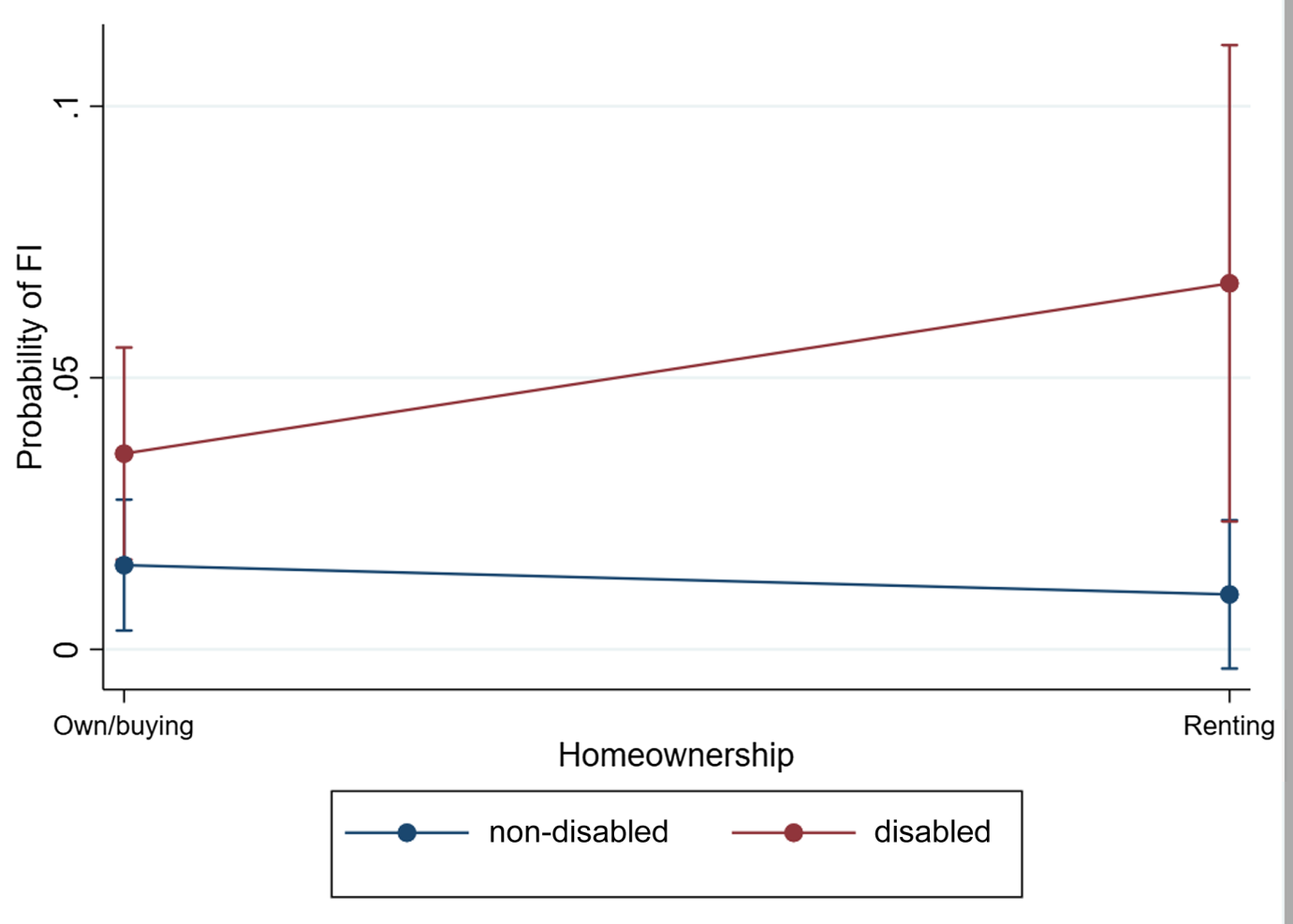


Note: Predicted probabilities from a logistic regression model adjusted for sex, ethnicity, presence of child(ren) in household, household income, household savings, and presence of partner (work status not adjusted for in 65+).

**Figure A4a:** Relationship between disability and food insecurity over age. In 2016 disability was defined as a ‘yes’ response to having a physical or mental health conditions or illnesses lasting or expected to last for 12 months or more and then respondents saying ‘a little’ ‘a lot’ response to the condition or illness reducing respondent’s ability to carry-out day-to-day activities. Only mobility, dexterity and vision difficulties were further specified.


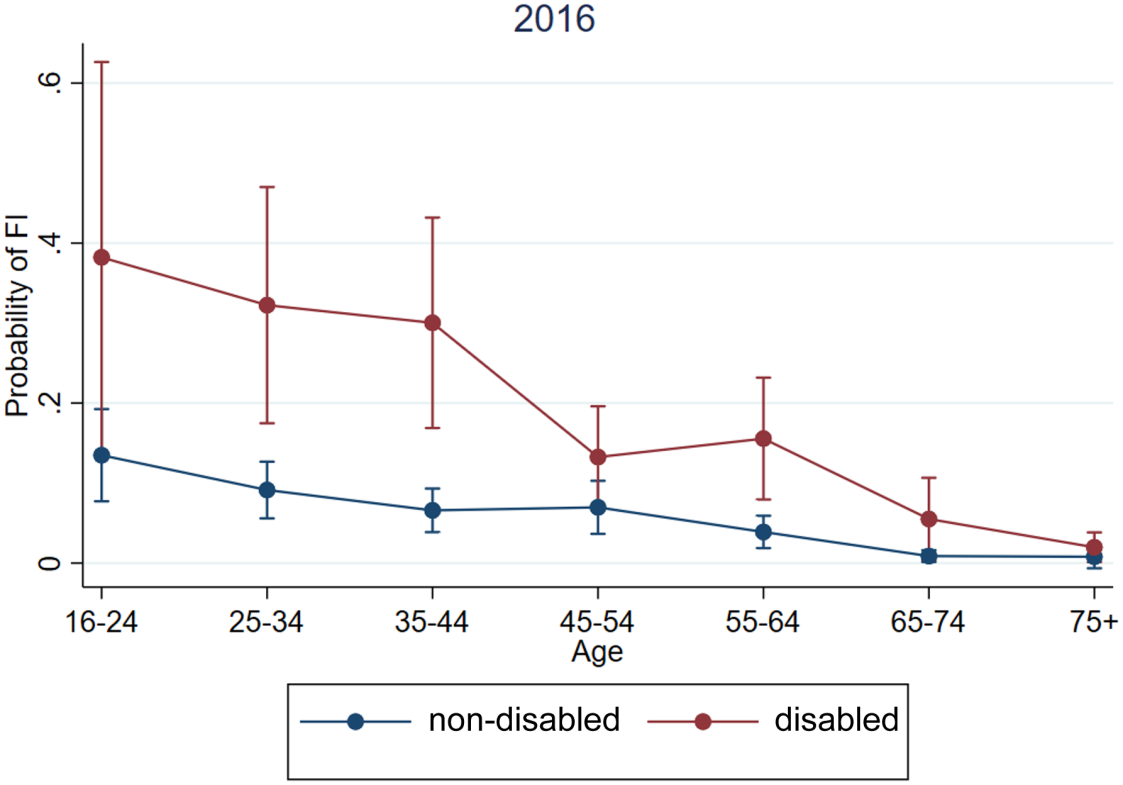


**Figure A4b:** Relationship between disability and food insecurity over age. In 2018 disability was defined as a ‘yes’ response to having a physical or mental health conditions or illnesses lasting or expected to last for 12 months or more. Respondents were then asked whether they were impacted in the following areas: Vision, Hearing, Mobility, Dexterity, Learning or understanding or concentrating, Memory, Mental health, Stamina or breathing or fatigue, Socially or behaviourally, Other.


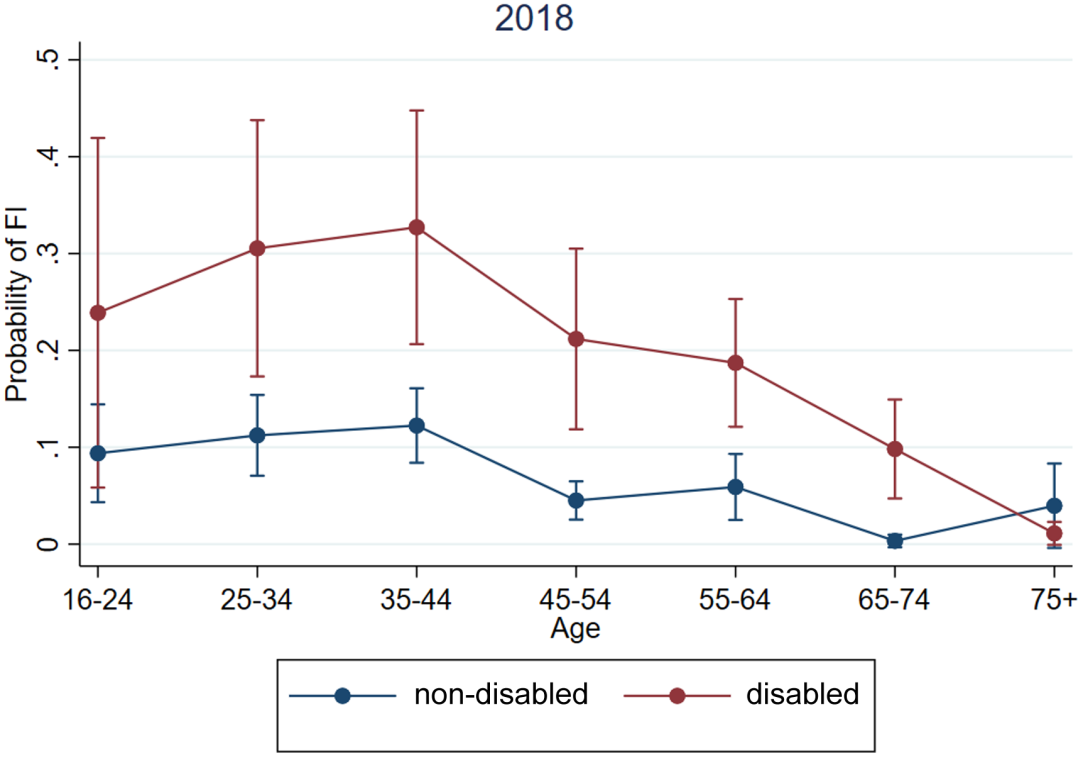

Supplement: Hadfield-Spoor et al. supplementary material [file S1368980024000570sup001.docx]
